# Nursing home contrasts: A critical discussion on harnessing reflection for dignified change

**DOI:** 10.1177/09697330251374177

**Published:** 2025-09-05

**Authors:** Trude Anita Hartviksen, Lisbeth Uhrenfeldt, Jessica Aspfors

**Affiliations:** 1 Center for Care Sciences, North, UiT, the Arctic University of Norway, Tromso, Norway; 2 Department of Health and coping, The Municipality of Vestvågøy, Leknes, Norway; 3 Department of Orthopedic Surgery, Kolding Hospital, Tromso, Denmark; 4 Department for Regional Health Research, Southern Danish University, Odense, Denmark; 5 Faculty of Education and Arts, Nord University, Bodø, Norway; 6 Faculty of Education and Welfare Studies, Åbo Akademi University, Turku, Finland

**Keywords:** dignity, dignified care, nursing homes, relatives, healthcare middle managers, critical discussion

## Abstract

The capacity of healthcare personnel to promote well-being and dignity among nursing home residents remains a topic of ongoing discourse. This critical discussion extends from a previously published research project on healthcare leadership. The results revealed that healthcare middle managers’ perceived lack of resources, trust, and collaboration hindered their capability for quality improvement. Our concern was reinforced when relatives expressed a willingness to accept undignified care due to resource constraints. In this discussion paper, we add to existing knowledge by critically discussing how nursing home relatives and managers share experiences and face the consequences of negotiating human dignity as both a fundamental right and moral obligation. Galvin and Todres’s existential theoretical framework is applied to understand dignified care and to reveal contrasts and overlaps within academic and public discourse. We argue that resource-limited healthcare, which generates undignified care, creates cross-pressure of suffering for relatives and managers. If both groups are incapacitated by this cross-pressure, knowledge mobilization and practice improvement stagnate. However, integrating measures into organizational quality systems to promote critical reflection among both groups as a tool for quality improvement may offer potential dignified change.

## Introduction

The lack of dignity in the care of older persons has emerged as a pressing issue, garnering significant concern in both academic literature^1^ and public discourse.^2^ Efforts to promote human dignity are widely recognized as essential for fostering a more equitable world and are acknowledged as a vital aspect of sustainable development.^3,4^ From 2019 to 2023, the EU-funded Marie Sklodowska-Curie project, INNOVATEDIGNITY,^5^ successfully educated and trained 15 early-stage researchers to become leaders in innovative, dignity-related projects. This ambitious initiative fostered critical thinking and inspired the authors of this paper to delve deeper into the complex concept of dignity. The resulting insights contributed to the advancement of a previously published three-part research project on healthcare middle managers (HMMs)^6–8^ incorporating fresh theoretical perspectives on the well-being and dignity of nursing home residents. These perspectives have not been previously presented, as they emerged through a reflective and iterative process of reviewing the results of the three earlier studies within a coherent and integrated critical discussion. This evolving discussion, presented in the current paper, has already influenced administrative and political decisions, suggesting that, if sustained, it could spark transformative change, ultimately improving the quality of life for nursing home residents.

## Background

The concept of dignity has faced criticism due to the complexity and challenges associated with its conceptualization and application. The criticism has led to the recognition of dignity as a multidimensional construct.^9^ In this discussion paper, dignity is understood as a fundamental aspect of human existence that encompasses self-worth, respect, and the intrinsic value of human beings.^10^ Safeguarding dignity is recognized as the core of a caring culture^11^ and is essential for vulnerable populations, such as nursing home residents.^10^ Nursing homes provide long-term care for older persons who can no longer live independently, offering personal care, nursing, and other healthcare services.^12^

Our previous research project adopted a Scandinavian perspective, where nursing homes are part of the welfare state. The facilities are publicly financed through taxes and residents’ deductibles and are generally operated by municipalities.^13^ The Dignity Guarantee, a key principle of the welfare state, is enshrined in regulations^14^ and legislation.^15^ This principle mandates treating all individuals with respect, preventing dignity violations, promoting equality and social justice, and ensuring access to basic needs to uphold human dignity.^14^ The guarantee is exemplified in the emphasis on body care as a core element of nursing, justified by its essential role in preserving the well-being and dignity of older persons.^16^

Nevertheless, challenges related to the well-being and dignity of older persons in nursing homes are well-documented, both in Scandinavia and internationally. Extensive research has shown that nursing homes often struggle to meet residents’ needs, align with expectations, and balance economic constraints. This situation has been described as a systemic failure^12^ and is expected to worsen due to demographic changes (an increasingly older population), resource shortages, and the need to strengthen knowledge-based services. Carcavilla-Gonzalez et al.^17^ have suggested addressing these challenges through political efforts, including deinstitutionalization, investment in interdisciplinary teams, and competence development in home-based services.

The challenges of translating policies on dignity and well-being into practice are well-documented and are largely attributed to a healthcare culture that impedes subjective, first-person perspectives. Western healthcare is often described as being shaped by national guidelines focused on measurable outcomes. Healthcare personnel embedded in this technically driven environment may find themselves trapped in a negative feedback loop, reinforcing the belief that speaking up is futile. This dynamic can lead to emotional consequences and a decline in the quality of care provided to residents.^18^ In the broader societal context, substantial research has demonstrated that care for older persons is often stigmatized and associated with low occupational status and professional denigration. Such conditions have been shown to undermine care quality and contribute to high staff turnover and difficulties in recruitment.^19^ Transforming nursing practices requires the proactive engagement of nurses in critical reflection on their lived experiences within the professional domain.^20^ Other factors influencing the negotiation of complex human organizations include hierarchies based on profession, expertise, gender, or ethnicity.^21^

A systematic review^22^ revealed that older persons often experience their dignity being undermined as they become socially invisible and unrecognized. Older persons’ understanding of quality care is multidimensional, emphasizing the importance of healthcare personnel identifying their individual priorities and tailoring services accordingly.^23^ The safeguarding of dignity for persons with dementia has been shown to depend on both formal and informal caregivers. Factors contributing to undignified care include stigmatization, objectification, resource scarcity, time constraints, alienation, and misunderstandings.^24^ Ageism is considered a significant threat to older persons’ well-being.^25^ In contrast, dignified care is primarily rooted in relational aspects, such as personalization, respect, attentiveness, and encouragement.^24^

Relatives of nursing home residents—including significant others, next of kin, family members, and other loved ones—expect quality care, efforts to safeguard residents’ human dignity, cooperation, honesty, and mutual trust.^26^ Although relatives are recognized as playing a crucial role in safeguarding residents’ well-being, quality of life, and sense of identity, they are often excluded from planning and decision-making processes. This exclusion is considered a contributing factor to the systemic failure.^27^

Balkin et al.^28^ explored well-being and dignity among nursing home residents, advocating for epistemological and ontological reflection by rethinking old age through the lens of Galvin and Todres.^10^ This theoretical framework has previously been presented as a practical and effective guide for enhancing the human aspects of care.^29^ While ethical education and reflection are recommended as ongoing processes in nursing homes, these areas are often underprioritized. Further research is needed to examine both the ethical competence of individual healthcare personnel and the organizational cultures in which they operate.^30^

The starting point for this discussion was a critical hermeneutic analysis of the development of leadership capacity and capability among HMMs in nursing homes.^6^ This research project was subsequently expanded to include relatives’ experiences regarding the well-being and dignity of nursing home residents.^7,8^ In this paper, we present an extended critical discussion provoked by the overall results of the three studies that comprise this project.

### Summary of the three studies’ relevance to a novel discussion

The previous research project included five semi-structured focus groups involving seven HMMs and eighteen relatives from two publicly funded nursing homes in a rural Arctic municipality in Norway.^6–8^ The nursing homes reported staffing challenges, consistent with international demographic trends.^24^ The first of the three studies aimed to identify and critically discuss how the development of HMMs’ leadership capacity and capability influenced quality improvement in nursing homes. The results revealed that HMMs made deliberate efforts to mitigate the impact of complexity and resource limitations by supervising quality, engaging in knowledge development, and compensating for observed quality deficiencies.^6^ However, the study raised concerns as these efforts were found to occur within a contradictory context characterized by unclear frameworks and a lack of leadership support. Additionally, troubling results emerged regarding the roles of relatives in nursing homes. Although these results fell outside the initial study’s scope, they led to two subsequent secondary analyses.^7,8^

The second study aimed to critically discuss relatives’ experiences of influencing dignified care for nursing home residents. The results indicated that relatives attempted to prevent instances of missed care when dignity was at stake by either pointing out problems with healthcare personnel or addressing their loved ones’ needs themselves. Despite their good intentions, relatives often experienced alienation in their efforts to transform undignified practices into dignified care.^7^ While these experiences of alienation were beyond the scope of this study, the abundance and significance of the data prompted a new secondary analysis.^8^

The third study aimed to critically discuss relatives’ experiences of suffering when their next of kin resided in nursing homes within a rural Arctic context. The results revealed that relatives struggled to adapt to a dependent relationship with healthcare personnel and nursing home management. They experienced resignation and felt pressured to accept what they perceived as violations of their loved ones’ dignity, often becoming silent witnesses to missed care. Relatives’ experiences of suffering in their attempts to interact responsibly with healthcare personnel were conceptualized as cross-pressure.^8^

Galvin and Todres’s existential framework^10^ was employed in both secondary analyses to critically discuss the concept of dignity^7,8^ and has also informed the discussion presented in this paper. This discussion continued throughout and beyond the research project as consistent ethical challenges were identified across the studies. For instance, shared experiences and patterns of action emerged among relatives and HMMs in their encounters with missed care or indignity. Parallels were also drawn between this research project and broader international literature.

This ongoing critical discussion has informed the development of theoretical argumentation presented in this paper, in which this previous empirical work is further developed and connected to broader theoretical and societal questions. It aims to critically discuss how nursing home relatives and HMMs share experiences and face the consequences of negotiating human dignity as both a fundamental right and a moral obligation.

### Ethical considerations

The previous research project was conducted in accordance with the principles of the Helsinki Declaration, which continues to guide this present discussion paper. Prior to data gathering, participants were informed both orally and in writing about the purpose, the confidentiality of their data, and their right to withdraw at any time. Written consent was obtained, including permission for the empirical data to be used in future studies related to nursing home quality. Ethical approval from the Regional Committee for Medical and Health Research Ethics (REK) was deemed unnecessary (reg. no. 2018/1905). The project was registered with the Norwegian Centre for Research Data (NSD) (reg. no. 993360).

## Critical discussion

A third-level discourse, as described by Habermas,^31,32^ is introduced to expand the theoretical argumentation of the previous research project^6–8^ and to contribute to existing knowledge more broadly. Notably, the application of Habermas’s theory of communicative action to quality improvement in nursing practice has been previously explored,^20^ as well as the potential for nursing values to be emancipated from the dominance of economic considerations.^33^ Building on this foundation, we have drawn on Habermas’s conceptualization of communicative rationality. This approach transcends instrumental and strategic rationality by emphasizing dialogue aimed at achieving mutual understanding and agreement.^31^ We are inspired by Shabani’s^34^ operationalization of communicative rationality, which includes descriptions of how third-level discourse is grounded in shared norms and values and employs language not merely as a tool for individual goal attainment but also as a means to coordinate actions collaboratively.

Habermas’s concept of third-level discourse provides a structural framework that integrates ethical considerations, making it essential for facilitating democratic decision-making, fostering informed citizenship, and addressing complex societal challenges that cannot be resolved through purely instrumental or strategic approaches.^34^ In this paper, we elaborate on how ethical concerns can mobilize knowledge among relatives, healthcare personnel, managers, and decision-makers. We argue that such mobilization may have an emancipatory effect, contributing to political changes that improve conditions for nursing home residents.

Supported by this theoretical framework,^31,32^ we critically discuss contrasts in communication, power relations, and structures^31^ as they pertain to dignified care.^10^ This paper was developed through the authors’ engagement in a reflective, iterative process,^32^ reviewing the results of previous research to identify contrasts and overlaps with existing knowledge. For example, the first study showed that HMMs either pointed out concerns to health personnel or personally acted to address perceived lapses in dignity or missed care,^6^ and the second revealed that relatives assumed a similar role.^7^ The third study demonstrated how this role led to suffering among relatives, ultimately resulting in resignation.^8^ Upon revisiting the results from the first study,^6^ we identified previously unpublished data indicating that HMMs experienced similar forms of suffering. This result was therefore incorporated into the subsequent critical discussion. This iterative process—led and documented by the first author—involved thematic organization of prior results. Further interpretation was facilitated by Galvin and Todres’ existential framework^10^ and relevant existing knowledge, while contrasts and overlaps were identified drawing on Habermas.^31,32^ These elements subsequently informed the further third-level discourse within the author group. Issues of concern were critically discussed until consensus was reached. This collaborative process led to the development of three key insights, which will structure the following discussion.

## Human dignity as a fundamental right and moral obligation

The three key insights that emerged from the discussion are (1) resource-limited healthcare generates undignified care; (2) cross-pressure of suffering when observing undignified care; and (3) measures for critical reflection offer potential dignified change.

### Resource-limited healthcare generates undignified care

The first insight—resource-limited healthcare generates undignified care—prompted the critical discussions, when focus group participants, including both relatives and HMMs, shared descriptions of undignified care in nursing homes. Our concern deepened upon discovering that relatives appeared willing to accept such circumstances, attributing them to the municipality’s financial constraints, time limitations among healthcare personnel, and the overall lack of resources in nursing homes. This apparent acceptance stood in stark contrast to the principles of the Dignity Guarantee^14,15^ and even to the relatives’ own stated knowledge, values, and norms.

As researchers, we were alarmed to encounter a group of relatives who reported that they had never been consulted about their experiences with nursing home care. They shared several troubling examples of what they perceived as undignified, self-directed care.^7,8^ This feedback is problematic when considered alongside well-established knowledge regarding the importance of positive family involvement in improving overall nursing home quality^27^ and how its absence may signify systemic failure.^12,27^

Both relatives and HMMs reflected a shared understanding of dignity as a fundamental aspect of human existence, encompassing self-worth, respect, and the inherent value of human life.^10^ Their examples of undignified care overlapped^6–8^ and sharply contrasted with the objectives outlined in current regulations^14^ and legislation,^15^ particularly regarding breaches of individualized care and self-determination. Relatives frequently reported instances of coercion, medication mismanagement, and failures to ensure adequate nutrition or mealtime assistance. Some described feeling obligated to be present daily to ensure proper care. Additional observations included failures to maintain normal circadian rhythms, insufficient outdoor access, and neglect of hygiene support—despite these being fundamental components of nursing practice.^16^

HMMs expressed a lack of capability to prevent missed care, citing directives from top management to prioritize office work, manage sick leave, and adhere to budgetary constraints.^6–8^ These results align with previous research on how hierarchies influence decision-making in complex organizations.^21^ However, the results also revealed that participating relatives primarily expressed a sense of resignation and refrained from providing feedback to HMMs regarding their observations of undignified care.^8^ This insight contradicts existing knowledge, which suggests that relatives consistently voice expectations for dignified care in nursing homes.^26^ The inconsistency between the numerous examples of undignified care provided by both relatives and HMMs and the standards set by the Dignity Guarantee^14,15^ raises critical questions about the practical implementation of this Guarantee. These results contribute to descriptions of systemic failure in nursing homes.^12^

Considering these arguments, Galvin and Todres’s description of dignity^10^ provides a valuable framework for critically discussing relatives’ efforts to influence dignified care in nursing homes. Our results demonstrate how relatives actively participate to ensure that their loved ones receive dignified care. However, the discussion takes a different direction when relatives describe the need to repeatedly remind healthcare personnel of residents’ needs or even personally intervene to compensate for undignified care. These results align with statements from HMMs, who reported that they constantly monitor and compensate for lapses in dignified care.^6^ This overlap suggests that both relatives and HMMs assume similar supervisory roles in nursing homes to uphold residents’ dignity. Notably, we have not found comparable descriptions in existing literature, underscoring the need for further exploration of this phenomenon.

It can be considered a paradox that, while relatives sought to ensure dignified care, they inadvertently contributed to inequality, creating disparities between residents due to inconsistencies in active family involvement.^7^ Furthermore, the acceptance of undignified care, attributed to an acknowledgment of resource-limited healthcare, was found to generate suffering among relatives—caught in the continuum between residents’ dignity and indignity, as well as between their own feelings of well-being and suffering.^8^ Suffering was exemplified through the ways in which relatives described their observations of undignified care, including their resignation when attempting to monitor and compensate for it without perceiving any lasting improvement.^8^ These experiences contribute to Galvin and Todres’s descriptions of suffering,^10^ contextualizing it within a nursing home setting. This led us to the next insight of discussion: cross-pressure of suffering when observing undignified care.

### Cross-pressure of suffering when observing undignified care

The second key insight—cross-pressure of suffering when observing undignified care—evolved throughout the discussions based on the descriptions of suffering provided by both relatives and HMMs. Initially, the concept of cross-pressure was used to depict the suffering experienced by relatives.^8^ However, HMMs described similar experiences, albeit framed as conflicting practices. HMMs shared accounts of being caught between a rock and a hard place, navigating the demands of clinical practice with residents and healthcare personnel while contending with pressure from top management and politicians. These challenges are well-documented for HMMs^11^ and provide a theoretical foundation for identifying cross-pressure among both relatives and HMMs, arising from significant contrasts between values and demands.

Our suggestion of cross-pressure as a new factor within Galvin and Todres’s framework on suffering^10^ acknowledges the significant role relatives play in nursing homes, capturing the complexity of their experienced suffering. Their descriptions of suffering cannot be easily categorized into the six existential factors outlined by Galvin and Tordres,^10^ as they are deeply interconnected. Relatives reported suffering when observing undignified care as a cross-pressure between their individual values, preferences, actions, and decision-making, which conflicted with family resources, traditions, values, age, culture, and gender. Additionally, they described a cross-pressure between institutional policies, management practices, recruitment challenges, and the individual values and decisions of healthcare personnel, perceiving these various demands as contradictory.^8^ These experiences of cross-pressure diverge from Havreng-Théry et al.,^26^ who found that relatives viewed themselves as a strength in nursing home care.

HMMs’ experiences of conflicting practices were driven by competing needs and demands from various stakeholders, compounded by resource scarcity, and contributed to the development of a culture that also perpetuated suffering for HMMs. Although we did not initially link these experiences to cross-pressure, this interpretation emerged through subsequent critical discussions. HMMs described struggles with resource limitations, unclear frameworks, role conflicts, and a lack of trust, collaboration, and support for leadership development. These results contextualize existing knowledge about systemic failure in nursing homes by illustrating the complexity of addressing these dynamics.^12^

**Figure 1.**
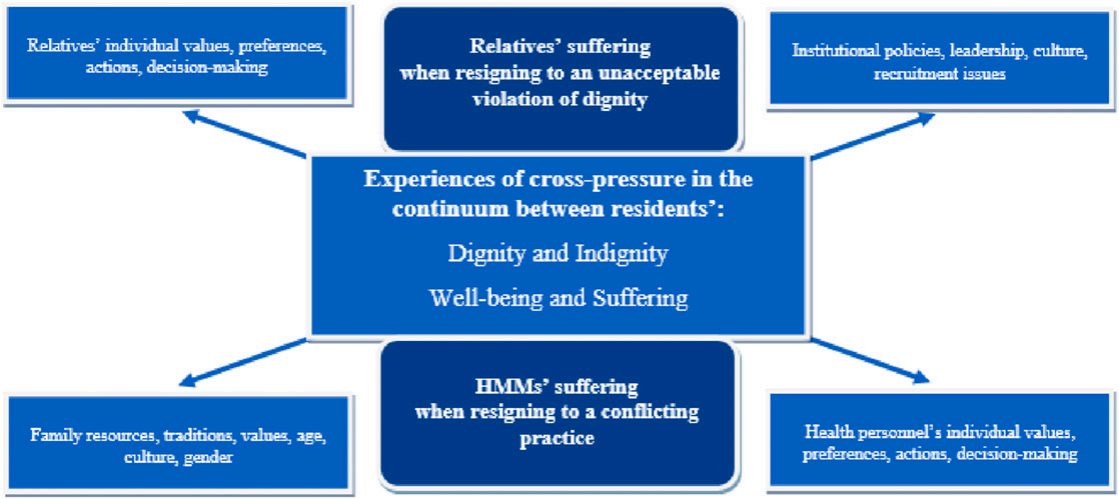
Experiences of cross-pressure in the continuum between residents’ dignity and indignity—well-being and suffering—among relatives and HMMs.

HMMs expressed both anger and resignation in response to their contradictory demands.^6^ One example involved their perceived need to be clinically present to ensure that residents received dignified care,^14,15^ while simultaneously facing pressure from top management to spend more time in the office to reduce costs.^6^ Through the lens of Galvin and Todres’s framework,^10^ we developed new theoretical arguments linking this form of suffering to both personal identity as HMMs and the intersubjective relationship between leadership and residents. Consequently, we refined our earlier visualization of cross-pressure,^8^ as shown in its updated form in Figure 1.

Although relatives and HMMs shared similar descriptions of their experiences with cross-pressure, relatives expressed their emotions with stronger language and provided more examples than HMMs. This observation suggests that relatives may experience stronger emotional reactions than HMMs when confronted with the same instances of suffering related to undignified care. We have discussed whether this disparity may be attributable to the professional role adopted by HMMs in focus groups, in contrast to the personal role embodied by relatives. Healthcare stakeholders are typically categorized according to their roles—such as manager, healthcare personnel, resident, or relative—each associated with distinct perceptions of reality.^18^ Known differences in language choices reflect these roles, as the ethos of HMMs traditionally centers on responsibility, respect, recognition, and awareness of attitudes and actions concerning patients and healthcare personnel.^11^ While older persons’ expressions of anger can signify a perceived threat to their dignity,^22^ missed interactions have primarily been attributed to individual challenges faced by relatives.^12^ However, other studies have reported similarities in observations across different stakeholder groups.^24^ In our research project, descriptions of cross-pressure included relatives’ expressions of anger and resignation when witnessing situations that contradicted the Dignity Guarantee.^14,15^ These results contrast with existing knowledge, which suggests that caring for nursing home residents inherently involves caring for their family members.^12^

Our critical discussion is further enriched by results from the research project, which included a few statements suggesting that undignified care was not solely attributable to resource constraints but also to challenges related to unethical attitudes, values, and a lack of competence among healthcare personnel.^8^ Interestingly, this aspect introduced an element to the discussion that the relatives appeared reluctant to explore further. Both resource limitations and challenges stemming from healthcare personnel’s individual attributes are well-recognized as factors influencing the quality of care. Undignified care in nursing homes is also associated with cultural factors, such as stigmatization and the low status attributed to work with older persons.^19^ Residents with dementia have been shown to be particularly vulnerable to depersonalization.^23^ However, these contributing factors were not mentioned by participants in our study—neither by HMMs nor by relatives—as potential explanations for the undignified care they described. Instead, one relative suggested that such incidents were understandable given the challenges of working with persons with dementia.^8^ This absence of broader explanations invites reflection on whether indignity is more readily accepted due to residents’ age, illnesses, and cognitive impairments—a dynamic that aligns with theoretical discussions of ageism.^25^ The first and second insights led us to our third key insight, which forms the current focus of our discussion: measures for critical reflection offer potential dignified change.

### Measures for critical reflection offer potential dignified change

In this theoretical discussion of practice-related experiences concerning dignity and dignified care in nursing homes, we comply with the established ethical responsibility for researchers to ensure that new knowledge contributes not only to academic publications but also to tangible change and improvement in practice. Dignity is not merely a philosophical phenomenon; it carries practical significance.^22^ The third key insight in this critical discussion posits that measures for critical reflection offer potential dignified change.

Research has demonstrated that systematic reflection on care values in nursing homes increases value awareness among all stakeholders.^11^ In our previous research project, participants reported that the focus group discussions contributed to personal growth and prompted them to question their own acceptance of undignified care.^6–8^ The development of leadership capacity among HMMs was grounded in critical reflection, enabling a deeper understanding of the complex and ongoing processes within nursing homes. HMMs’ capacity was strengthened through ongoing guidance and an empowering approach to healthcare personnel, rooted in dignified values. However, role conflicts, resource shortages, and a lack of trust and collaboration hindered their capability to successfully improve dignified care and allocate time for critical reflection.^6^ Previous research emphasizes the importance of critical reflection as an ongoing practice in nursing homes,^30^ particularly for enhancing nursing practice.^20^ Critical reflection is particularly essential when working with older persons,^9^ yet it is frequently deprioritized. Under time pressure, nurses may prioritize routine tasks over meaningful engagement with nursing home residents, which can undermine dignified care.^30^

The participating relatives emphasized the importance of ongoing critical reflection. Their reflections led them to repeatedly attempt to change practices to ensure that their loved ones received dignified care. However, our results revealed that relatives often experienced their critical feedback as being perceived negatively by both healthcare personnel and HMMs, rarely resulting in improvements in dignified care.^7^ This insight evolved throughout the research project. Despite relatives’ accounts of residents experiencing embodied and interpersonal indignity^10^ and the cross-pressure they endured, relatives often adapted to a passive role in nursing home settings. They described feeling compelled to suppress their critical reflections after witnessing undignified care, even when these conflicted with their own values and the principles of the Dignity Guarantee.^14,15^ We have previously referred to this emotional suppression as a “pressure cooker” of unresolved feelings.^8^

Several participants in our previous research project described the critical reflection that occurred within the focus groups as emancipatory, noting that it gave them the courage to cope with their challenging situations. This applied to both relatives and HMMs.^6^ Although the relatives consistently downplayed their observations of indignity and offered explanations as to why such situations had to be accepted, they also affirmed that indignity could not be tolerated in any form. This duality characterized the dynamic back-and-forth movement in the focus groups, which aligns with Habermas’s concept of communicative rationality.^31^

**Figure 2.**
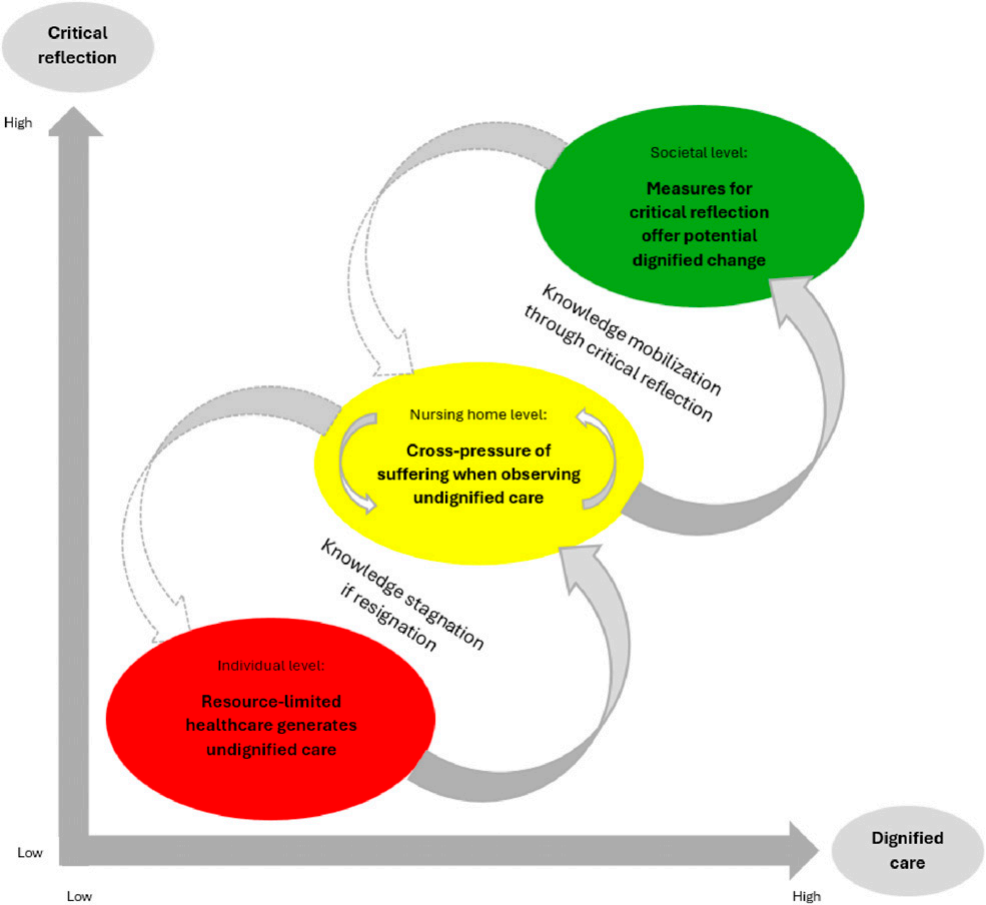
Activated critical reflection offers the potential to foster meaningful changes toward dignified care.

Reflection is recognized as a precondition for the expression of dignity.^35^ In this discussion paper, we build on this understanding by suggesting that critical reflection holds significant emancipatory potential for fostering change toward dignified care. Figure 2 illustrates this dynamic: The *X*-axis represents the level of dignity in care (low to high), while the *Y*-axis represents the degree of critical reflection in nursing homes (low to high). The three key insights are visually connected by arrows forming a spiral, symbolizing how the process can lead to either knowledge mobilization or knowledge stagnation, depending on the effectiveness of critical reflection.

In the figure, we have marked the first insight from this critical discussion—resource-limited healthcare generates undignified care—in red to symbolize the danger it poses for nursing home residents. The upward arrow from the first to the second insight indicates how observations of undignified care create cross-pressure for both relatives and HMMs in nursing homes. Internal arrows illustrate how measures of critical reflection can activate these cross-pressures, creating potential for change rooted in dignified values. This insight is therefore marked yellow.

The downward arrow from the second to the first insight signifies that non-activated critical reflection—or resignation—leads to knowledge stagnation, whereby both cross-pressure and undignified care remain unchanged. Conversely, the upward arrow from the second to the third insight demonstrates how activated critical reflection can facilitate knowledge mobilization. We argue that this has the potential to foster meaningful change, which is why it is marked in green. This potential served as the foundation for extending the critical discussion presented in this paper, while also taking the conclusions further into municipal systems, as we will elaborate on in the next paragraph.

However, the downward arrow from the third insight points to a contradiction: If activated critical reflection is not supported by a quality system that facilitates improvements toward dignified care for all residents—including those without family members present—critical reflection could inadvertently increase disparities. This, in turn, may exacerbate the experienced cross-pressure and intensify feelings of guilt or pangs of conscience.

### Implications for practice, policy, and theory

The theoretical argumentation presented in this paper has been shared and further discussed with healthcare management in several Norwegian municipalities, initiating a process of knowledge mobilization with practical implications. For example, these arguments have prompted critical reflection and a reassessment of what constitutes dignified care, particularly in relation to the quality of care in nursing homes. This reassessment has identified areas for improvement, which are currently being addressed through initiatives focused on competence development, quality improvement, and reorganization. We suggest that this knowledge has the potential to influence changes in practice, provided there is adequate support for knowledge transfer and critical reflection. Such support must be secured through clear routines within quality systems, ensuring that the facilitation of critical reflection includes not only healthcare personnel and management but also residents and relatives.

Our results reveal a significant gap between nationally mandated policies and their implementation in nursing home practices. Even in countries with legislation aimed at ensuring dignity, both relatives and HMMs report situations where vastly different realities unfold. Paradoxically, during municipal elections, politicians consistently promote nursing homes as a secure option for the care of older persons. This contradiction between academic knowledge and political discourse underscores the urgent need to develop clear, theoretically grounded national clinical guidelines that effectively operationalize regulatory requirements for practical application, regardless of political shifts.

In selecting new solutions for healthcare for older persons, it will be necessary to develop competence not only among healthcare personnel but also among citizens, as well as administrative and political decision-makers. This requires ongoing processes across multiple decision-making levels to develop trust, competence, and collaboration. The critical discussion presented in this paper demonstrates how theoretical development and practical advancements can generate knowledge mobilization; theory is strengthened by practice, and practice is enhanced by theoretical insights. This represents an example of how practice-based research, through its applicability and theoretical elevation, can provide direct and meaningful implications for residents in nursing homes.

## Conclusion

This ongoing critical discussion began with an exploration of leadership development among HMMs in nursing homes, before an evolving concern moved us onto relatives’ statements and subsequently revisiting HMMs’ perspectives. We conclude that our contribution to theoretical argumentation lies in uncovering previously unexplored connections between these actors, revealing both contrasting and overlapping experiences with undignified care in nursing homes. Both groups reported resource shortages; however, only relatives—despite employing stronger language in their description—expressed a willingness to accept undignified care as a consequence of these shortages. Both relatives and HMMs described experiences in which they either sought to alert healthcare personnel to deficiencies in care or chose to compensate for these deficiencies themselves, thereby adopting similar supervisory roles.

Despite the relatives’ purported acceptance, both they and the HMMs portrayed various forms of suffering based on their observations. This suffering was conceptualized as perceived cross-pressure for both groups, albeit with somewhat differing characteristics. Relatives described cross-pressure as a sense of being unwelcome guests in the nursing home, where they had to balance their own perspectives with those of their family and weigh institutional demands against individual needs. These demands were often described as contradictory, leading us to adopt the metaphor of a “pressure cooker” from our previous research project. HMMs described cross-pressure as the navigation of conflicting practices involving resource scarcity, unclear frameworks, role conflicts, and a lack of trust, collaboration, and support.

We observed a lack of critical reflection among both relatives and HMMs. When critical reflection occurred among relatives, the subsequent failure to address their suggestions often led to knowledge stagnation rather than mobilization. We suggest that facilitating critical reflection among relatives, healthcare personnel, leaders, and policymakers has the potential to promote knowledge mobilization, fostering meaningful changes and more dignified care in nursing homes. However, successful implementation requires clear routines embedded within quality systems, ongoing processes to build trust and competence, and collaboration across decision-making levels.

## Data Availability

Data sharing is not applicable to this article, as no new data were created or analyzed in this study.*
